# A possible correlation between facet orientation and development of degenerative cervical spinal stenosis

**DOI:** 10.1186/s12891-024-07279-3

**Published:** 2024-02-27

**Authors:** Haimiti Abudouaini, Junsong Yang, Kaiyuan Lin, Yibing Meng, Hong Zhang, Sibo Wang

**Affiliations:** 1 Department of Spine Surgery, Honghui Hospital, Xi’an Jiaotong University, Xi’an, China; 2 Department of Ultrasound Medical Center, Honghui Hospital, Xi’an Jiaotong University, Xi’an, China

**Keywords:** Degenerative cervical spinal stenosis, Facet joint, Sagittal orientation, Anteroposterior diameter

## Abstract

**Background:**

Previous studies have demonstrated the relationship between sagittal facet orientation and cervical degenerative spondylolisthesis. However, the associations between facet orientation and cervical spinal stenosis (CSS) have rarely been studied.

**Methods:**

One hundred twenty patients with CSS (CSS group) and 120 healthy participants (control group) were consecutively enrolled. The cervical facet angles and anteroposterior diameter (A-P diameter) of spinal canal at each subaxial cervical levels were measured using axial magnetic resonance imaging. The intersection angle of the midsagittal line of the vertebra to the facet line represents the orientation of the facet joint.

**Results:**

The facet angles on the right side at C2- C3 and C3-C4 in CSS group and at C2- C3 in control group had significantly higher values than those of the other sides. Besides, the facet angles and A-P diameter of spinal canal in CSS group were significantly smaller than those in control group at all levels (*p* < 0.05).

**Conclusions:**

Our study demonstrated that patients with CSS have smaller axial cervical facet joint angles compared to the healthy individuals. Further studies are needed to elicit the specific underlying mechanism between sagittalization of the cervical facet joints and the pathology of CSS.

## Introduction

In contrast to intervertebral discs, facet joints are the sole synovial joints found in the spine. It has been documented that facet joint degeneration tends to occur prior to disc degeneration in approximately 20% of spinal segments [1]. The presence of facet joint abnormalities has been linked to various spinal disorders, including degenerative scoliosis [2], lumbar instability [3], disc herniations [4], and bone hyperplasia [5]. Over time, this deterioration weakens the core muscles, resulting in the development of neck pain and low back pain [6, 7]. Several recently published studies have outlined the relevance of increased sagittal orientation of the facet joints in the cervical degenerative spondylolisthesis (DS) [8, 9]. Regarding the mechanism of cervical DS, different theories have been proposed. One such theory is that the mechanism of cervical DS is different from that of lumbar DS; specifically, cervical DS is caused by intervertebral disc degeneration and is propagated by the facet joints and ligaments [10], another scholar conclude that mechanism for cervical DS development is indeed analogous to lumbar DS, in which hypertrophic degeneration of the facet joints results in altered cervical mechanics and secondary spondylolisthesis [11].

Recently, a significant association between facet orientation in lumbar segments and degenerative lumbar spinal stenosis (DLSS) was found in the several studies [12–16]. Miyazaki et al. [13] observed the lumbar spine canal diameter by using kinetic magnetic resonance imaging (MRI) and found that patients with sagittally oriented facets have narrow osseous canals. Liu et al. [14] also reported that facet sagittal orientation is a independent risk factor for the development of DLSS.

Despite previous studies demonstrating the correlation between sagittal facet orientation and cervical degenerative spondylolisthesis [8, 17, 18], there is a scarcity of research examining the associations between facet orientation and cervical spinal stenosis (CSS). Given the paucity of clinical data in this area, a retrospective analysis was conducted to enhance our comprehension of the etiology and progression of CSS. This study aimed to compare the facet angles at C3-C7 measured in the transverse plane on MRI between patients with CSS and those without CSS. The anticipated outcomes of this study are expected to provide valuable insights and practical recommendations for the management of CSS patients in the foreseeable future.

## Materials and methods

### Participants

Patients who had cervical MRI between March 2016 and March 2020 at our institution were retrospectively screened for the enrollment. All personal information was de-identified and analyzed anonymously. Measurements were taken by two individuals using the Phoenix PACS software (version 3.20.34233; Phoenix PACS GmbH, Freiburg, Germany) and recorded to the nearest 0.1 mm. One hundred and twenty patients with cervical spinal stenosis were enrolled as the study group (CSS group). The inclusion criteria were Torg-Pavlov Ratio (TPR) < 0.75 on sagittal T2W scans, and typical clinical symptoms, such as inability to button shirt or walking difficulties. Exclusion criteria were scoliosis, spondylolisthesis, ossification of posterior longitudinal ligaments, rheumatoid arthritis, spinal infection, spinal tumors, previous fractures, or previous spine surgery. Patients with facet tropism were also excluded. Facet tropism was defined a more than 7 difference in bilateral angles with respect to the axial plane [9, 10].

The control group also included 120 patients who performed cervical X-ray, Computed Tomography (CT), and MRI at outpatient clinic in our hospital. There were no cervical spinal stenosis (MRI_TPR_ > 0.75), spinal cord compression, trauma, discitis and previous cervical surgery in these patients.

### Imaging measurements

**Fig. 1:**
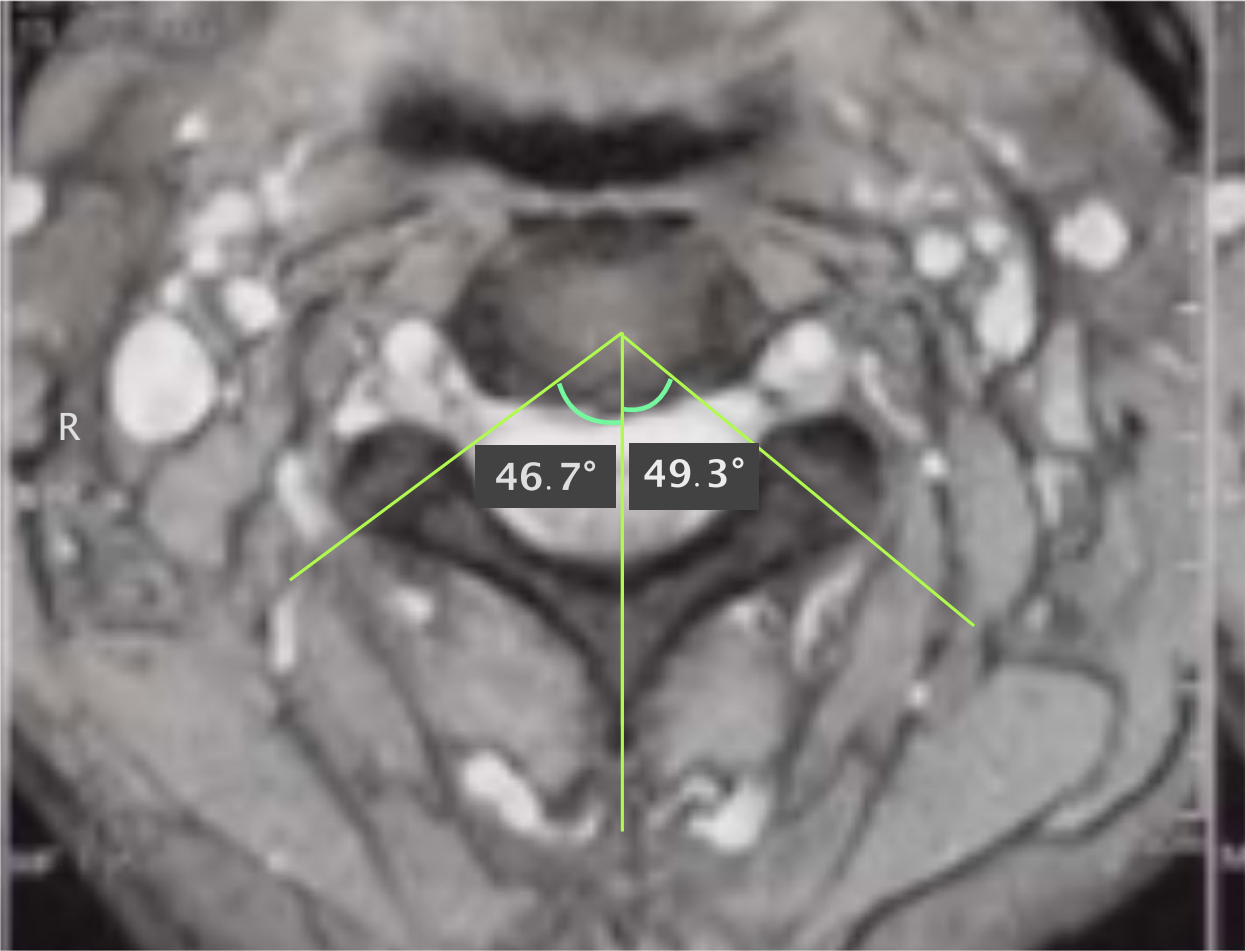
Measurement method of the facet joint angle and anteroposterior diameter (A-P diameter) of spinal canal in the transverse MRI image. A facet line was drawn connecting the anterior and posterior margins of each of the superior articular facet. Then, a mid-sagittal line was added that passed through the center of the base of the spinous process. The angle between the facet line and the midsagittal line was defined as the facet angle

**Fig. 2:**
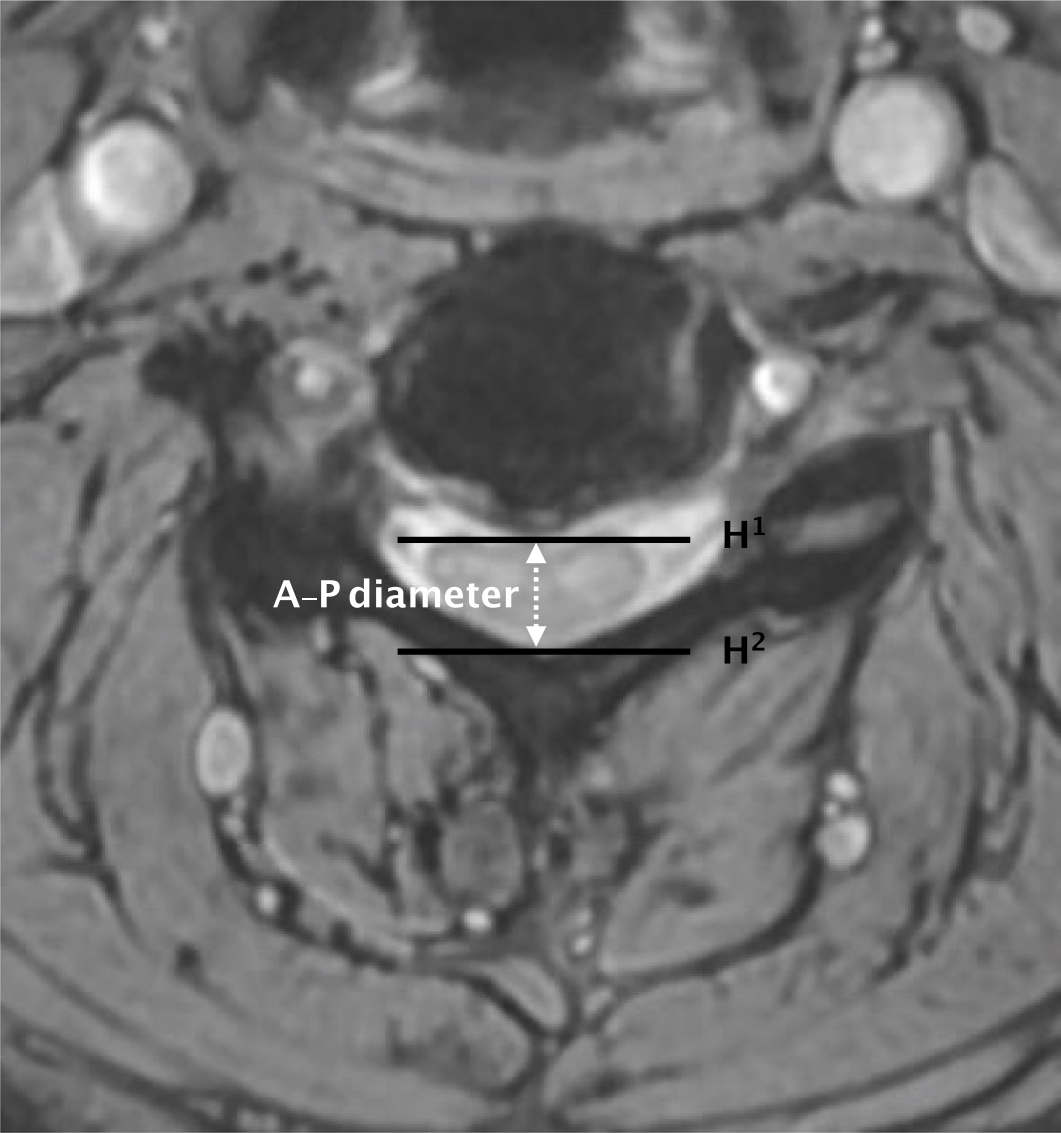
Cross section view of the cervical spinal cord measured on T2-weighted magnetic resonance images. A–P diameter was defined as the highest distance of spinal cord, H1 was the highest and H2 was the lowest points of the spinal canal

Following parameters were measured on axial MR images both in study and control group, respectively. Facet orientations at C2-C3, C3-C4, C4-C5, C5-C6 and C6-C7 were determined from MR images. The T2-weighted axial images were obtained parallel to the end plate. The T2-weighted axial images with the best view of facet joint in the caudal of intervertebral disc were chosen for the facet angle measurement. The facet angle was measured as described in previous studies [11, 12]. The line drawn through the center of the disc and the center of the base of the spinous process was defined as the reference plane. The line drawn between the anteromedial and posterolateral edges of the bilateral superior articular facets was defined as the facet line. The angle between the two lines was defined as the facet joint angle (Fig. 1). Anteroposterior diameter (A–P diameter) was also measured in the transverse MRI image as described in previous studies [13, 14] (Fig. 2).

### Statistical analysis

All the statistical analysis was performed by SPSS 25.0 software (SPSS Inc., Chicago, IL, USA). Continuous data were demonstrated as means ± standard deviation. The independent samples t test was used to compare the differences in facet angle and A-P diameter between different gender and patients in CSS and control group. For all analyses, a two-sided probability value below 0.05 was considered to indicate statistical significance.

## Results

### Patient demographic data

The study consisted of a total of 240 patients, with 120 patients (70 men and 50 women) in the CSS group and 120 healthy participants (65 men and 55 women) in the control group. The average age of the CSS group was 52.34 ± 11.28 years, while the average age of the control group was 53.98 ± 11.03 years. Statistical analysis revealed no significant differences between the two groups in terms of age and BMI (Table 1, *P* > 0.05).

**Table 1:** Comparison of the average facet angle between male and female

	CSS group			Control group		
	Female	Male	*P*	Female	Male	*P*
No.^a^	50	70	-	55	65	0.515
Age^b^	52.95 ± 12.03	51.66 ± 10.45	0.547	53.27 ± 11.52	54.58 ± 10.64	0.518
	(34, 78)	(33, 77)		(35, 79)	(34, 78)	
BMI^b^	23.56 ± 2.21	23.64 ± 2.56	0.856	22.98 ± 2.35	23.01 ± 2.65	0.958
	(19.36, 27.66)	(18.37, 28.52)		(19.61, 27.55)	(18.44, 28.34)	
C2-C3^b^	50.49 ± 9.32	52.90 ± 7.68	**0.035**	53.42 ± 7.57	54.81 ± 5.62	0.116
	(26.5, 62.3)	(28.6.5, 61.5)		(29.2.5, 62.7)	(30.5, 63.8)	
C3-C4^b^	49.77 ± 8.20	50.77 ± 8.73	0.265	54.88 ± 8.96	56.16 ± 6.68	0.209
	(31.7, 69.2)	(30.2, 70.1)		(32.4, 70.3)	(33.6, 74.6)	
C4-C5^b^	48.96 ± 6.92	48.76 ± 8.14	0.838	56.87 ± 6.09	56.47 ± 7.13	0.648
	(36.4, 69.6)	(34, 69.3)		(38.5, 69.6)	(40.5, 70.1)	
C5-C6^b^	52.71 ± 7.32	51.12 ± 8.40	0.130	55.68 ± 6.40	57.20 ± 6.38	0.069
	(38.6, 72.1)	(36.1, 70.3)		(40.9, 71.5)	(40.2, 72.9)	
C6-C7^b^	55.74 ± 9.37	56.39 ± 8.55	0.647	61.55 ± 7.40	62.92 ± 7.30	0.151
	(40.2, 71.9)	(40.2, 71.9)		(45.2, 74)	(43.8, 75.4)	

^a^Chi-square test; ^b^Independent sample t—test

### Comparison of facet angle

**Fig. 3:**
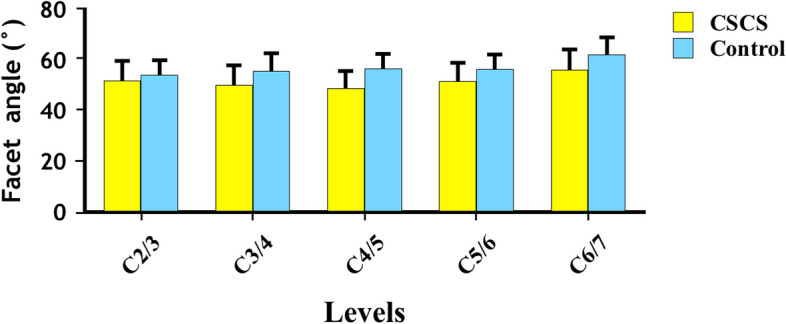
The bar chart of facet angle distribution at different levels

The results are summarized in Tables 1 and 2 and Fig. 3. In CSS group, the facet angle at C2/3 level was 50.49 ± 9.32° in female patients and 52.90 ± 7.68° in male patients (*p* = 0.035). No significant difference between female and male was found in the other level in CSS group and at all levels in control group (*p* > 0.05). The facet angles on the right side at C2- C3 and C3-C4 had significantly higher values than those of the other side for the CSS group, and facet angles on the right side at C2- C3 also significantly higher than those of the left side in control group. The facet angles in CSS group were significantly smaller than those in control group at all levels (*p* < 0.05). Besides, the facet angles and A-P diameter in axial position at all subaxial cervical disc level were significantly higher in control group than CSS group (*p* < 0.05, Table 3). However, no significant correlation was found between sagittal angles on each side and the canal AP dimensions in both groups (Table 4).

**Table 2:** Comparison of the facet angle between CSS and control group

	CSS (right)	CSS (left)	Control (right)	Control (left)	p	CSS (right)	Control (right)	CSS (right)	CSS (left)
						vs	vs	vs	vs
						CSS (left)	Control (left)	Control (right)	Control (left)
C2-C3^a^	52.11 ± 7.8	51.68 ± 9.1	54.73 ± 6.1	53.54 ± 7.1		**0.010**	**0.037**	**0.001**	** < 0.001**
C3-C4^a^	50.49 ± 9.2	50.01 ± 7.9	56.08 ± 7.5	55.06 ± 7.8		**0.025**	0.313	**0.001**	** < 0.001**
C4-C5^a^	49.89 ± 7.5	47.80 ± 7.7	57.38 ± 6.4	55.93 ± 6.8		0.718	0.217	** < 0.001**	** < 0.001**
C5-C6^a^	52.49 ± 8.4	51.08 ± 7.5	56.81 ± 6.7	56.19 ± 6.2		0.399	0.914	** < 0.001**	** < 0.001**
C6-C7^a^	56.11 ± 8.8	56.12 ± 9.0	62.23 ± 7.6	62.34 ± 7.6		0.696	0.376	** < 0.001**	** < 0.001**

^a^Independent sample t—test

**Table 3:** Comparison of A-P diameter in transverse position between the groups

	CSS group	Control group	*p*
C2-C3^a^	8.05 ± 1.06 mm	11.25 ± 0.82 mm	** < 0.001**
C3-C4^a^	7.21 ± 0.89 mm	11.08 ± 0.68 mm	** < 0.001**
C4-C5^a^	8.52 ± 0.96 mm	11.06 ± 1.60 mm	** < 0.001**
C5-C6^a^	8.29 ± 0.65 mm	11.38 ± 0.65 mm	** < 0.001**
C6-C7^a^	8.65 ± 1.32 mm	11.97 ± 0.93 mm	** < 0.001**

^a^Independent sample t—test

**Table 4:** Correlation between sagittal angles and canal AP dimensions in different both groups

		CSS group	Control group
C2-C3^a^	Right	0.016 (0.860)	-0.155 (0.091)
	Left	0.092 (0.319)	0.001 (0.989)
C3-C4^a^	Right	0.159 (0.083)	0.030 (0.747)
	Left	-0.121 (0.187)	0.134 (0.143)
C4-C5^a^	Right	-0.200 (0.058)	-0.40 (0.665)
	Left	-0.169 (0.064)	0.16 (0.886)
C5-C6^a^	Right	-0.017 (0.854)	-0.040 (0.655)
	Left	0.027 (0.769)	0.016 (0.866)
C6-C7^a^	Right	-0.233 (0.010)*	0.015 (0.872)
	Left	-0.919 (0.036)*	0.075 (0.417)

^a^Independent sample t—test;  * Indicates statistically significant differences (*p*<0.05)

## Discussion

Cervical spinal stenosis (CSS) is often a result of degenerative cervical spondylosis that may cause chronic compression of the cervical spinal cord, eventually leading to dysfunction [19, 20]. These age-related degenerative changes including intervertebral disc disease, vertebral remodeling, hypertrophy and/or ossification of spinal ligaments, and spondylolisthesis [20]. Although it was reported that sagittal orientation of the facet joints is a risk factor of cervical degenerative spondylolisthesis [8–10, 21], and to our knowledge, the direct relationship between sagittal orientation of the facet joints and CSS not yet been explored.

The present study demonstrated that the cervical facet orientations changed from level to level. The facet joint angles were more vertically oriented in the lower region of the subaxial spine, especially at the C6/C7 level, which is in agreement with some previous studies [21, 22]. Rong et al. [21] and Pal et al. [22] also reported the increasing facet joint angles from C2-C3 to C6- C7 in horizontal plane. In contrast, Ebraheim et al. [23] evaluated the cervical facet joints using 41 cervical spines from C3 to C7, and they found that facet joint angle was decreased from 51.7° at C2/3 level to 44.2° at C6/7 level. This difference may be due to factors such as different measurement means, patient positions, differences between reference planes, and ethnic differences. Different to previous studies, in this study we also found that the facet angle of female patients was significantly smaller than that of male patients at C2-C3 in the CSS group.

The facet joints play a prominent role in stabilizing the motion segment in flexion, extension, and axial rotating restriction [24]. In 1988, PAL and his colleagues conducted a cadaveric study and found that 36% of the total load applied to the superior articular surfaces of the axis vertebra is transmitted through the anterior column formed by bodies and intervertebral discs, and 32% each through the two posterior cervical columns formed by the articular processes [25]. Another biomechanical study also confirmed that the facet joint bears 33% of the dynamic load and 35% of the static load of the spine [26]. There are some differences in the anatomical characteristics of facet joints in each segment. For example, the superior and inferior articular facets lie in the same vertical line at the C3-C6 level. However, at the C2 and C7 levels, superior and inferior articular facets do not lie in the same vertical line. Because of this, the loads diffuse into the laminae at C2 and C7 levels while being transmitted from the superior to the inferior articular facets [27].

In recent years, facet tropism become a research hotspot in those studies that related to cervical facet joints. To analyze the relationship between facet orientation and CSS more accurately, we excluded the patients who has a more than 7 difference in bilateral angles with respect to the axial plane [26, 28]. The relationship between facet angles and the development or progression of CSS is not necessarily straightforward and is likely influenced by multiple factors. Gaining an understanding of how smaller facet angles impact load bearing in the cervical spine is of utmost importance for clinical relevance. However, due to the retrospective nature of this clinical study, it is challenging to elucidate the precise mechanism involved. Nonetheless, it has been reported that smaller facet angles may result in a more uniform distribution of load, potentially alleviating stress on structures and offering a biomechanical advantage. In contrast, the concentration of stresses on specific regions due to smaller facet angles may potentially contribute to the degenerative processes associated with CSS [29]. Considering the present study's results, it is plausible to hypothesize that smaller facet angles could lead to the concentration of stresses on particular regions in the cervical spine, thereby potentially contributing to the degenerative processes associated with CSS. Further investigation is necessary to comprehend the intricate nature of the potential influence of smaller facet angles on load bearing in the cervical spine and their significance in the development or progression of CSS. Although the current literature offers some understanding of the biomechanical aspects, future research is imperative to establish more definitive associations between facet angles, load distribution, and the pathophysiology of CSS.

The findings of our study indicate that there is a statistically significant decrease in facet angles among females in the CSS group at the C2-C3 level, which aligns with previous literature. In a study conducted by Rong et al. [30], radiological observations on a sample of 200 subjects revealed a higher prevalence of facet degeneration in females specifically at the C2-C3 level. Previous research has demonstrated that facet joint angles have a significant impact on the distribution of loads within the spinal column [31]. Alterations in these angles can affect the levels of stress and strain experienced by neighboring structures [32, 33]. It is plausible that smaller facet angles could contribute to heightened stress on adjacent structures, thereby potentially exacerbating the advancement of spinal stenosis. The recognition of sex-specific disparities in facet angles underscores the significance of incorporating gender as a determinant in the assessment and management of CSS. Subsequent investigations ought to delve into the fundamental mechanisms that contribute to these variations, potentially encompassing hormonal, genetic, or developmental factors.

The anteroposterior diameter of cervical spinal canal (A–P diameter) in MRI has been demonstrated that is an effective method to reflect cervical spinal stenosis [34–36]. The standard of CSS using A–P diameter shows marked ethnic differences in previous studies. Different to western countries, which suggested that cut-off value of cervical spinal canal diameter was 13 mm [32, 35], it was reported A–P diameter < 11 mm is more advisable to indicate cervical spinal stenosis in our region11. In their study, A–P diameters on transverse image were 11.11 mm–12.43 mm and 6.68 mm–10.76 mm in the healthy group and spinal stenosis group respectively. In our study, the average A-P diameter at all levels is between 11-12 mm in the control group and 7-9 mm in CSS group. The etiology of variation of facet orientation is remains unclear. Some scholars theorize that facet orientation results from cervical spine degeneration [8, 9, 37], while some believe that it is caused by developmental alterations [38].

The research findings presented in this study have the potential to provide clinical applications for both patients and physical therapists. Specifically, gaining an understanding of the relationship between facet orientation and the development of degenerative cervical spinal stenosis could have significant practical implications. One such implication is the identification of preventive strategies, as the correlation between facet orientation and spinal stenosis development could pave the way for implementing measures aimed at preventing its occurrence. Besides, physical therapists can administer patient-specific interventions, including targeted exercises or lifestyle modifications, which can be investigated to alleviate the advancement of degenerative changes. By incorporating their understanding of facet orientation into treatment plans for individuals with degenerative cervical spinal stenosis, physical therapists can customize interventions that may enhance the efficacy and personalization of rehabilitation strategies.

The most significant limitation of this study is that we analyzed the facet orientation only in the transverse plane. If facet orientation in sagittal and coronal plane can also be analyzed, a more comprehensive and accurate relationship between facet orientation and CSS could be obtained. Besides, although imaging parameters were measured by two individuals, there also exists the possibility of a measurement error. As this was a retrospective study, we are unable to ascertain the cause-effect relationship between facet orientation and CSS. Another limitation of the present study is the absence of age-specific subgroup analysis. Moreover, the present study did not include the indicators that can reflect the extent of cervical cord compression among CSS patients. In our forthcoming research, we will examine the correlation between facet orientation and cervical cord compression by employing the Torg-Pavlov ratio and compression ratio for each individual cervical level.

## Conclusion

Our study demonstrated that patients with CSS have smaller axial cervical facet joint angles compared to the healthy individuals. Multicenter, large sample biomechanical studies are needed to elicit the specific underlying mechanism between sagittalization of the cervical facet joints and the pathology of CSS.

## Data Availability

The datasets used and/or analysed during the current study are available from the corresponding author on reasonable request.
